# Molecular and Cellular Mechanisms of Antitumor Immune Response Activation by Dendritic Cells

**Published:** 2016

**Authors:** O. V. Markov, N. L. Mironova, V. V. Vlasov, M. A. Zenkova

**Affiliations:** Institute of Chemical Biology and Fundamental Medicine, Lavrentieva Ave., 8, Novosibirsk, 630090 , Russia

**Keywords:** dendritic cells, subsets, antigen presentation and cross-presentation, proteasome, tumor immunosuppression

## Abstract

Dendritic cells (DCs) play a crucial role in the initiation and regulation of the antitumor immune response. Already , DC-based antitumor vaccines have been thoroughly explored both in animal tumor models and in clinical trials. DC-based vaccines are commonly produced from DC progenitors isolated from peripheral blood or bone marrow by culturing in the presence of cytokines, followed by loading the DCs with tumor-specific antigens, such as DNA, RNA, viral vectors, or a tumor cell lysate. However, the efficacy of DC-based vaccines remains low. Undoubtedly, a deeper understanding of the molecular mechanisms by which DCs function would allow us to enhance the antitumor efficacy of DC-based vaccines in clinical applications. This review describes the origin and major subsets of mouse and human DCs, as well as the differences between them. The cellular mechanisms of presentation and cross-presentation of exogenous antigens by DCs to T cells are described. We discuss intracellular antigen processing in DCs, cross-dressing, and the acquisition of the antigen cross-presentation function. A particular section in the review describes the mechanisms of tumor escape from immune surveillance through the suppression of DCs functions.

## INTRODUCTION

Today, methods based on the activation of the immune system are of particular importance in cancer therapy. Dendritic-cell- (DC)-based vaccines capable of triggering and maintaining a tumor-specific T and B cell immune response stand out among various approaches [1]. DCs are professional antigen-presenting cells (APCs) the main function of which is to capture foreign antigens and process and present them on the cell surface in complexes with major histocompatibility complex (MHC) class I and II molecules to naive T cells. This interaction results in the maturation and activation of tumor- specific cytotoxic T lymphocytes (CTLs) capable of migrating to tumor sites, identifying tumor cells, and destroying them. In addition, the interaction triggers a response by type 1 and 2 T helper cells (Ths), which stimulates the T and B cell arms of the antitumor immune response. Additional stimulation by DC-secreted cytokines promotes the proliferation of tumor-specific CTL clones. The challenge today is to develop DCbased vaccines for the effective treatment of cancers and overcoming tumor-induced immunodeficient conditions.

The tumor microenvironment is known to suppress the immune system, which enables that tumor to escape immune surveillance. The tumor and its microenvironment produce various chemokines and cytokines that inhibit the maturation of APCs and T cells, which finally leads to the suppression of the functional activity of the T cell arm of antitumor immunity. Immunosuppression caused by the action of substances secreted by the tumor environment leads to the failure of standard treatments for malignant tumors. Therefore, the development of antitumor therapies based on the activation of the immune system is topical today. DCbased vaccines are considered as one of the most effective ways to overcome immunodeficiency on the basis of body resources.

This review describes the origin of DCs, their subsets, the molecular and cellular mechanisms of DCbased antitumor immune response activation, and the resistance of the tumor and its environment to the ability of dendritic cells to suppress tumor growth.

## BASICS OF DC FUNCTIONING: THE RELATIONSHIP TO INNATE AND ADAPTIVE IMMUNITY

The main task of the DCs present in all body tissues is to recognize exogenous or endogenous pathogenic antigens (Ags) and transmit the received information to adaptive immunity cells (naive T cells) through the presentation of Ags in a complex with the MHC molecules on the DC surface.

DCs are key cells that interconnect ancient low-specific innate immunity and evolutionarily new, highly specific adaptive immunity. DCs originate from bone marrow progenitors that a are common to monocytes, macrophages, and granulocytes – the main cellular factors of innate immunity. DCs share the common properties of these cells; in particular, the ability of phagocytosis, i.e. to uptake solids (cells, apoptotic bodies, proteins, etc). Indeed, almost all innate immune cells, except eosinophils and natural killers (NKs), use phagocytosis as one of the important mechanisms for the destruction of targets (bacteria and foreign or self, infected or tumor cells) [2]. DCs use phagocytosis, along with pinocytosis and receptor-mediated endocytosis, to uptake Ags for subsequent processing and presentation.

Innate immune cells have a nonspecific mechanism of target recognition using receptors that identify not single molecules (Ag epitopes), as the T cells of adaptive immunity, but groups of molecules, reporting on the foreignness or aggressiveness of their carriers [3]. For example, the surface of most innate immune cells bears lectins that recognize the terminal sugar residues of proteoglycans. The cell surface of DCs also possesses a large amount of C lectins, in particular mannose receptors (CD206) that bind terminal mannose residues [4]. Mannose receptors are also widely expressed by macrophages.

Another property common to DCs and innate immune cells, namely phagocytes (monocytes and macrophages), is the DCs ability to present Ags in complexes with MHC molecules to lymphocytes. However, DCs, which are professional APCs, stimulate T cells 10–100 times more effectively than other APCs (monocytes, macrophages, B cells) [5-7]. Only DCs are able to cross-present Ags most effectively; i.e. to present exogenous Ags in complexes with MHC class I molecules to CD8+ T cells, triggering an Ag-specific response by CTLs [8]. In addition, only DCs can present Ags to the naive T cells in lymphoid organs [9].

Another cellular factor of innate immunity is NKs that have a lymphocytic origin but differ from adaptive immune lymphocytes by a more primitive recognition mechanism and the only way of destroying target cells through perforin-dependent cytolysis involving perforin and granzyme [3]. DCs were shown to closely interact with NKs, stimulate NK proliferation and cytokine production, and also increase NK cytotoxicity. Activated NKs, in turn, play an important role in the elimination of immature tolerogenic DCs. On the other hand, NKs can induce DC maturation and affect the polarization of T cell responses. After recognizing a target, NKs secrete the tumor necrosis factor α (TNF-α) and interferon-γ (IFN-γ) that promote DC maturation and polarization of the T helper type 1 response (Th1 response). Furthermore, these cytokines enhance cross-presentation of Ags by dendritic cells to T cells. Thus, the relationship between DCs and NKs is of great importance in developing an effective tumor-specific adaptive immune response [10].

**Fig. 1:**
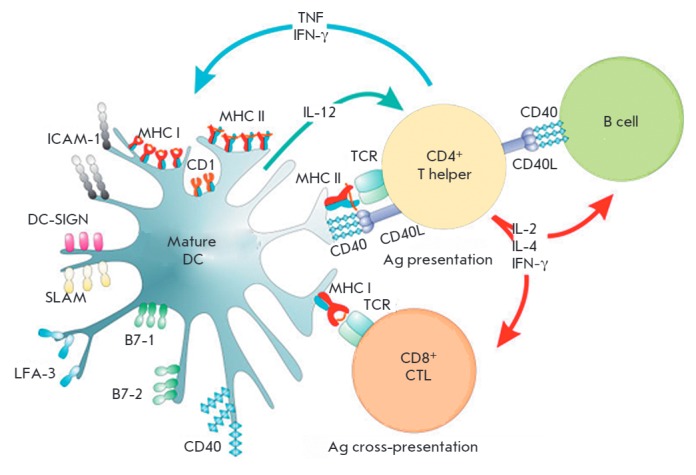
Interaction between DCs and CD4^+^ and CD8^+^ T cells [12].

To generate an antigen-specific adaptive immune response, immature DCs leave the bone marrow and migrate with the blood flow to peripheral tissues. There, DCs uptake foreign or self Ags, process, and expose Ags on the cell surface in complexes with MHC class I and II molecules. At the same time, DCs in peripheral tissues are affected by pathogenic agents and/ or inflammatory cytokines, which leads to DC maturation. Mature Ag-loaded DCs migrate via afferent lymphatic vessels to the lymph nodes, where they interact with naive CD4^+^ and CD8^+^ T cells [11, 12] (*Fig. 1*).

Upon interaction with DCs, naive T cells can differentiate into antigen-specific effector T cells with different functions. For example, CD4^+^ T cells can become type 1, 2, and 17 T helper cells, as well as regulatory T cells (Treg). Their main functions are to stimulate cytotoxic T cells, activate the B cells producing antibodies under their control, regulate the autoimmune and pro-inflammatory responses, and suppress the functions of other lymphocytes, respectively. Naive CD8^+^ T cells differentiate into CTLs that can specifically recognize and destroy tumor cells [13]. Therefore, DCs can, both directly and indirectly, specifically trigger, program, and regulate the T and B cell antitumor immune responses.

**The origin and subsets of DCs**

DCs are a heterogeneous cell population originating from a dedicated hematopoietic lineage of bone marrow progenitors [14]. There are several DC subsets differing in origin, phenotype, localization, migration pathways, functions, and, as a result, impact on innate and adaptive immunity [15]. These subsets can be grouped into two main groups: conventional DCs (cDCs) and plasmacytoid DCs (pDCs).

**DC precursors (pre-DCs)**

**Fig. 2:**
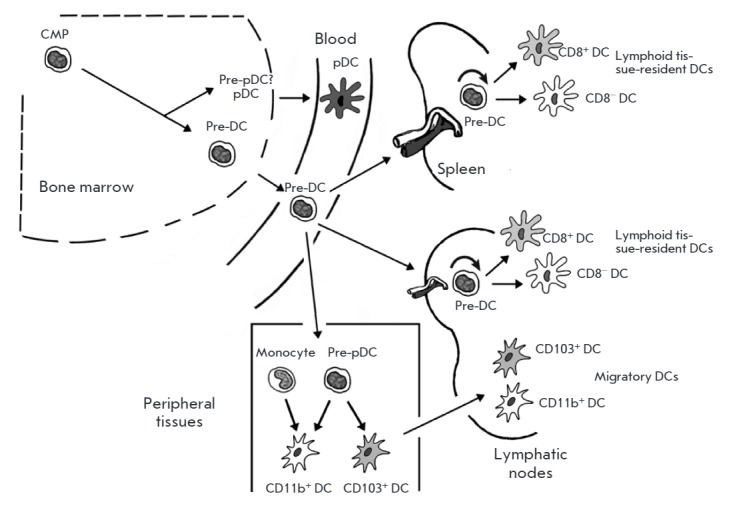
DC progenitors [16].

Pre-DCs are believed to originate from bone marrow precursors that lose, as they mature, the potential to develop into other cell types. This process is called commitment. The earliest committed pre-DCs are clonogenic common myeloid progenitors (CMPs), found in both mice and humans [16] (*Fig. 2*), that give rise to erythrocytes, granulocytes, megakaryocytes, monocytes, macrophages, DCs, and pDCs [17, 18].

Precursors of cDCs (pre-cDCs) with a Lin^-^CD11c^+^MHC II^+^ phenotype leave the bone marrow and travel with the blood to lymphoid organs, where they differentiate into lymphoid tissue-resident CD8^+^ and CD11b^+^ cDCs. They also occur in non-lymphoid organs, such as the liver, kidneys, lungs, and intestines, where they give rise to CD103^+^ and CD11b^+^ cDCs [19, 20]. Therefore, pre-cDCs are immediate cDC progenitors that permanently migrate from the bone marrow to the periphery to differentiate into the cDCs of peripheral tissues and resident DCs of lymphoid organs.

Langerhans cells are different from other DC subsets, because they self-renew independently of the bone marrow and differentiate from the precursors that entered the skin before birth [21]. However, these cells can develop from blood monocytes under inflammatory conditions, when the Langerhans cell population is very depleted [22].

**DC subsets**

*Plasmacytoid DCs*. pDCs are a small DC subset (0.3– 0.5% of human peripheral blood cells or mouse lymphoid organ cells) that share a similar origin with, but a different life cycle than, conventional DCs. pDCs accumulate mainly in the blood and lymphoid organs and migrate to the lymph nodes via the bloodstream [14]. A low expression level of MHC class II and co-stimulatory molecules was detected in pDCs. The CD11clowCD11b– CD45R/B220^+^ phenotype is typical of mouse cDCs [23, 24], and the Lin–CD11c–CD123(IL-3 Rα)+ phenotype is typical of human cDCs [25]. Most pDCs develop from common bone marrow preDCs (CDPs) with both a dendritic cell and lymphoid potential [26].

pDCs are called cells that produce type I interferons (IFN-α/β), because they secrete large amounts of IFN-α/β upon interaction between pathogenic nucleic acids and the Toll-like receptors (TLR3, TLR7, TLR8, and/or TLR9) expressed in pDCs [27-29]. In this case, a protective immune response is induced because IFNs-α/β enhance the cross-presenting ability of conventional DCs and activate immune cells, such as B and T cells and NK cells. Therefore, activated pD play an important role in innate and the adaptive immune responses [30].

Normally, mouse pDCs are localized in the lymphoid organs and blood, as well as in the liver, lungs, and skin. In humans, pDCs are found not only in the liver and blood, but also in lymphoid organs. They can migrate from the lymphoid organs through the bloodstream to the T cell zones of secondary lymphoid tissues and to the splenic marginal zone. In pathological conditions, pDCs leave the bone marrow, organs, or bloodstream and infiltrate inflamed tissues, where they interact with alarm signals (foreign Ags, pathogenic agents, etc.) and release large amounts of type I interferons [31].

*Conventional DCs*. Conventional DCs (cDCs) include all DCs, except plasmacytoid DCs. They can be found in most lymphoid and non-lymphoid tissues. cDCs can find damaged tissues, capture foreign or self Ags, and process and very efficiently present antigens to T cells. Therefore, cDCs can induce immunity in any foreign Ags entering tissues and trigger tolerance to self Ags.

cDCs constitutively express the hematopoietic markers CD45, MHC II, Flt3, and CD11c and lack the lineage-specific markers of T and B cells, natural killers, granulocytes, and erythrocytes [14]. According to their localization, conventional DCs can be classified into migratory non-lymphoid cDCs and lymphoid tissue-resident cDCs that never leave lymphoid organs.

**Fig. 3:**
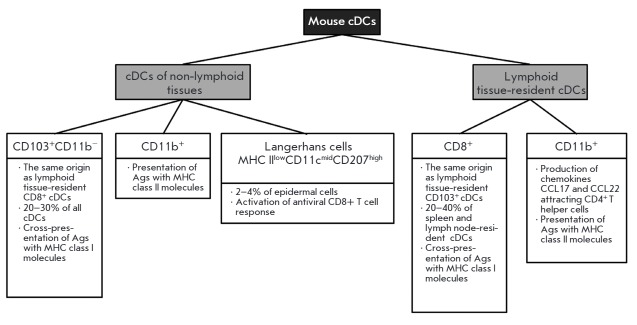
Subsets of mouse conventional DCs.

*Mouse conventional DCs. *In non-lymphoid tissue, cDCs account for 1–5% of the cells, depending on a particular organ, and consist of two subsets: CD103^+^CD11b^-^ and CD11b^+^ cDCs (*Fig. 3*). CD103^+^CD11b^-^ cDCs reside in most connective tissues. These are the main APCs that can more effectively, compared to other DC subsets, cross-present Ags to naive T cells [32] (*Fig. 3*). Both non-lymphoid tissue and lymphoid tissue-resident CD11b+ cDCs play the major role in the presentation of Ags with MHC class II molecules [33] (*Fig. 3*).

The third cDC subset, Langerhans cells, is presented in the epidermal skin layer. They account for 2–4% of the total amount of epidermal cells [34] and are characterized by MHC II^low^CD11^cmid^CD207^high^. Langerhans cells can trigger an antiviral CD8^+^ T cell response against various viral pathogens, except cytolytic viruses, such as herpes simplex and vaccinia viruses, because they possess the ability to induce the apoptosis of DCs, including Langerhans cells [35].

Resident cDCs of lymphoid organs consist mainly of two subsets: CD8^+^ and CD11b^+^ cDCs [36] (*Fig. 3*). CD8α^+^ DCs account for 20–40% of the cDCs of the spleen and lymph nodes. CD11b^+^ DCs prevail among lymphoid-resident cDC populations in all lymphoid tissues, except the thymus. These cells produce high levels of CD4^+^ T cell attractant chemokines CCL17 and CCL22 [14].

**Fig. 4:**
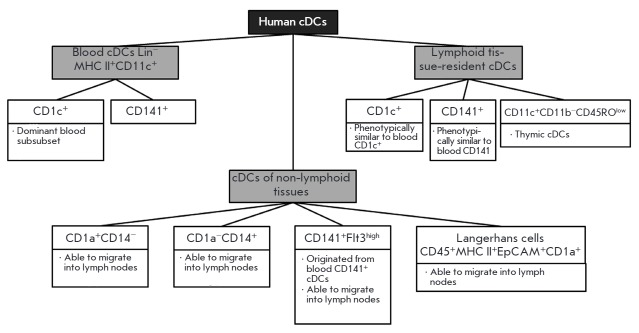
Subsets of human conventional DCs.

*Human conventional DCs*. The main difference between human and mouse cDCs is associated with the spectrum of surface markers. Human cDCs are divided into non-lymphoid tissue, blood, and lymphoid tissue- resident cDCs (*Fig. 4*). Human blood cDCs have the Lin^-^ MHC II+CD11c^+^ phenotype and are present in two subsets expressing non-overlapping markers: CD1c (BDCA1) or CD141 (BDCA3). The dominant peripheral blood DC subset is represented by CD1c^+^ cells, while CD141^+^ DCs form a minute population [14] (*Fig. 4*).

Non-lymphoid tissue cDCs include CD1a^+^CD14^-^ DCs, CD1a^-^CD14^+^ DCs [37], and a separate CD141^+^- Flt3high DC subset originating from peripheral blood CD141^+^ DCs [38]. Non-lymphoid tissue cDCs also include Langerhans cells expressing the markers CD45, MHC II, epithelial cell adhesion molecules (EpCAMs), langerin CD207, and CD1a [14] (*Fig. 4*).

Lymphoid tissue-resident cDCs consist of CD1c^+^ and CD141^+^ cDC subsets similar to blood DCs [38]. Lymph node cells also include MHC II^high^CD11^cmid^EpCAM^+^CD1a^+^, EpCAM^-^CD1a^+^, and CD206^+^ cells that are classified as migratory Langerhans cells, migratory dermal CD1a^+^ DCs, and dermal CD14^+^ DCs, respectively [39] (*Fig. 4*). Most human thymus cDCs have the CD^-^ 11c^+^CD11b^-^CD45RO^low^ phenotype and lack the myeloid markers presented on CD141^+^ DCs.

## FUNCTIONS OF DCS

**MHC class II antigen presentation by DCs**

**Fig. 5:**
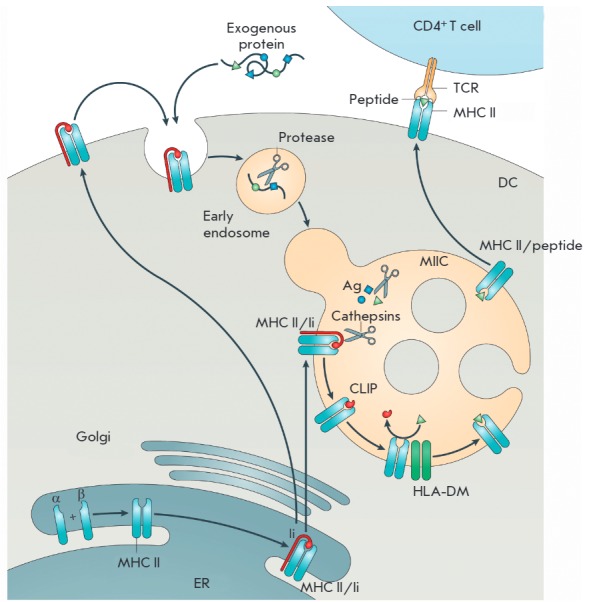
Presentation of exogenous antigens by DCs with MHC class II molecules [41].

Professional APCs (DCs, macrophages, and B cells) are characterized first of all by a high expression level of MHC class II molecules on the cell surface. Virtually all DC subsets are able to uptake exogenous Ags, process and present them in complexes with MHC class II molecules to CD4^+^ T cells, and trigger T helper immune responses of different types. For effective activation of a T helper response, DCs require, in addition to MHC II–Ag complexes, the presence of co-stimulatory and adhesion molecules (CD80, CD86, CD40, etc.) on the cell surface, as well as the synthesis of cytokines, such as IL-12, IFN-γ (Th1 response), IL-4 (Th2 response), or IL-23 (Th17 response) [40] (*Fig. 1*, *Fig. 5*).

*Fig. 5* depicts the presentation of exogenous Ags by DCs with MHC class II molecules. The MHC class II molecule is a heterodimer composed of two homogeneous peptides, the α- and β-chains, that are assembled in the endoplasmic reticulum (ER) and are attached to the invariant chain (Ii) (*Fig. 5*). MHC II/Ii complexes are transported to late endosomes, called MHC II compartments (MIIC). The transport is regulated by two dileucine motifs which are located on the cytoplasmic terminus of the invariant chain and are recognized by the sorting adaptors AP1 (a *trans*-Golgi network adaptor) and AP2 (a plasma membrane adaptor). The AP2-dependent endocytic endocytic transport pathway of MHC class II molecules from the plasma membrane to MIIC prevails in immature DCs, whereas the AP1-dependent transport from the *trans*-Golgi network is typical of mature DCs [41].

In MIIC, the invariant chain is cleaved from the MHC class II molecule by proteases cathepsin S and L, with the class II-associated Ii peptide (CLIP) remaining in the MHC class II peptide-binding groove. MHC class II molecules need a chaperone protein, H2-DM in mice or HLA-DM in humans, to exchange CLIP for a high-affinity antigenic peptide. To present Ags with MHC class II molecules, DCs use the vacuolar Ag processing pathway, where captured proteins are cleaved into peptides by lysosomal proteases.

The resulting MHC II/peptide complexes are transported in vesicles to the plasma membrane via fast microtubule transport involving motor proteins dynein (inward transport) and kinesin (outward transport), as well as via slow transport with actomyosin motor proteins.

The efficiency of Ag presentation with MHC class II molecules is inversely related to (i) the susceptibility of protein Ags to degradation and (ii) the concentration and activity of proteolytic enzymes in late endosomes. DCs differ from other phagocytic cells (e.g., macrophages) by a significantly lower expression level of lysosomal proteases and a reduced level of proteolytic activity. This is related to the high pH level of endosomal compartments, which is due to the low activity of V-ATPase and increased activity of NADPH-oxidase 2 [42].

**MHC class I Ag cross-presentation**

**Fig. 6:**
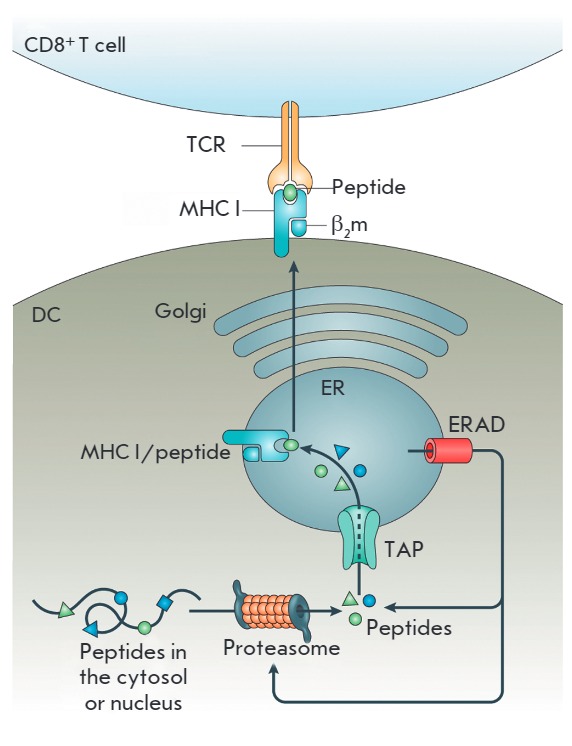
Cross-presentation of exogenous antigens by DCs with MHC class I molecules [41].

Cross-presentation is the presentation of exogenous Ags with MHC class I molecules, which is necessary to trigger a cytotoxic CD8^+^ T cell response (*Fig. 6*). DCs are unique APCs, because only they can cross-present Ags to naive CD8^+^ T cells [8]. This ability is essential for immune surveillance and enables the immune system to identify the tumors and viruses not infecting DCs. It should be noted that not all DC subsets have the ability of effective cross-presentation. In mice, the most effective DCs are migratory CD103^+^CD11b^-^ and lymphoid tissue-resident CD8^+^CD11b^-^ DCs [32]; in humans – CD141^+^ DCs [14].

MHC class I molecules are expressed by all nuclear cells. The main function of MHC class I molecules in cells is the presentation of endogenous AGs to the immune system to send a signal that this is not a foreign cell, but the organism’s cell. The MHC class I molecule is a heterodimeric protein consisting of a polymorphic heavy chain and a light chain called β2-microglobulin. The heavy chain polymorphism provides a variety of peptide-binding sites in MHC class I molecules, which enables MHC class I molecules to recognize unique antigenic peptides due to the differences in the anchor residues to which peptides dock [41].

MHC class I molecules accumulate in the ER and stay there due to the interaction with chaperone proteins, such as calnexin, calreticulin, ERp57, PDI, and tapasin, before binding peptides. MHC I heterodimers are unstable and readily dissociate under physiological conditions in the absence of a suitable peptide.

Processed antigenic peptides for cross-presentation are transferred by TAP proteins to MHC class I molecules in the ER. The peptide-binding site of MHC class I molecules accommodates 8 to 10 residue peptides, depending on the MHC haplotype. Peptides bind to the anchor sequences of MHC class I molecules mainly via N- and C-terminal amino acid residues, as well as via the side chains of some intramolecular residues [43]. Binding of a peptide to a MHC class I molecule leads to the stabilization of the interaction between MHC class I heavy and light chains and the release of chaperones. After this, the fully assembled MHC class I/peptide complex can leave the ER for presentation on the cell surface. This mechanism prevents the transport of “empty” MHC class I molecules to the plasma membrane to interact there with exogenous Ags. Peptides and MHC class I molecules that are not bound to the ER are returned to the cytosol for degradation [44].

**Ag processing and MHC class I complex formation**

There are two major mechanisms of Ag processing during cross-presentation: vacuolar and cytosolic. They can act either separately or simultaneously, depending on the type of cross-presented Ag. *Vacuolar Ag processing pathway. *In the vacuolar Ag processing pathway, the cross-presented Ags are processed and bind to MHC class I molecules inside endosomes/ phagosomes. One of the mechanisms suggests the involvement of the chaperone CD74 in the transport of newly synthesized MHC class I molecules from the ER to the endocytic compartments of DCs [45]. In the phagosome, Ag processing for cross-presentation involves cysteine protease cathepsin S [46]. In addition, the synthesis of cross-presented peptides during the cytosolic Ag processing pathway involves insulin-regulated aminopeptidase IRAP that is similar to the aminopeptidases ERAP1 and ERAP2 of the ER [47, 48].

*Cytosolic Ag processing pathway. *The cytosolic pathway plays a major role in the processing of Ags in DCs [46]. It was demonstrated that blockage of the vacuolar Ag processing pathway weakly inhibits cross-presentation and is even capable of strengthening it, while inhibitors of the proteasome and protein transport to the Golgi complex (lactacystin and brefeldin A, respectively) completely suppress the DC ability to cross-present Ags to CD8^+^ T cells [49].

In the cytosolic pathway, Ags are transported from phagosomes to the cytosol, where they are processed by proteasomal proteolysis, like endogenous Ags, for cross-presentation. The mechanism of Ag transport from endosomes to the cytosol is not completely understood. The mechanism is supposed to involve the ER-associated degradation machinery (ERAD machinery), in particular its constituent proteins SEC61 and p97 [50]. Translocation of DC-captured Ags by mannose receptors is controlled by ubiquitination of cytosolic mannose receptor sites. The protein p97, which is an ATP-ase, is attracted to the endosome/phagosome membrane through the interaction with polyubiquitinated mannose receptors [51]. An alternative mechanism of the endosome-to-cytosol translocation of Ags may include simple endosomal membrane destabilization by reactive oxygen species that are efficiently produced in the endocytic compartments of DCs [52].

Ags translocated to the cytosol undergo proteasomal processing that involves both conventional proteasomes and immunoproteasomes [53, 54]. Antigenic peptides generated by the proteasome and/or immunoproteasome are transported by TAP proteins into the ER lumen, where the peptides are hydrolyzed by terminal aminopeptidase ERAP1 into peptides of a suitable length for loading onto MHC class I molecules and subsequent cross-presenting to CD8^+^ T cells.

**Cross-dressing by DCs**

Apart from direct presentation and cross-presentation of exogenous Ags, there is an additional mechanism of Ag presentation, called cross-dressing, when DCs acquire MHC I/ antigen peptide complexes from dead tumor cells. Cross-dressing is mediated by secreted exosomes and trogocytosis that is a process by which cells exchange by cell membrane and membrane protein fragments. This enables DCs to present directly captured Ags without further processing. Unlike Ag cross-presentation involving the activation of CD8^+^ CTLs against DC-processed peptides, cross-dressing promotes activation of CD8^+^ T cells specific to peptides generated by the tumor cell, which may enhance the antigen specificity of the antitumor immune response. Cross-dressing can involve CD8α^+^/CD103^+^ DCs, activated and naive CD8^+^ T cells, and CD8^+^ memory T cells [55-57].

**Induction of DC cross-presentation function**

The cross-presentation function is acquired at the last stage of DC maturation, upon stimulation by microbial products, e.g. TLR-ligands, or cytokines, e.g. the granulocyte macrophage colony-stimulating factor (GM-CSF). Therefore, effective cross-presentation is typical of peripheral DC subsets during inflammation and infection.

CD8^+^CD103^-^ DCs isolated from a normal mouse spleen proved ineffective in the cross-presentation of Ags with MHC class I molecules in a medium lacking TLR ligands and cytokines, but they effectively presented Ags with MHC class II molecules [58]. Additional activation factors were required to induce cross-presentation, which indicates that this property of CD8^+^ DCs may be regulated.

TLR ligands not only activate the maturation of CD8^+^ DCs, but also promote an increase in the level of co-stimulatory and adhesion molecules on the DC surface and can also affect the processing of Ags by CD8^+^ DCs and enhance the cross-presentation of Ags [58].

In addition to microbial products, such as TLR ligands, the cross-presentation function in early progenitors of CD8^+^ DCs can be induced by GM-CSF. Normally, GM-CSF is produced at a low level, but its production is dramatically increased in infection or inflammation. GM-CSF-based induction of the cross-presentation function of DCs is not accompanied by an increase in the expression of standard DC activation markers (MHC II, CD80, CD86, or CD40), but the expression of CD103, a key marker of migratory DCs, is increased.

The mechanism of cross-presentation induction in CD8^+^ DCs under the influence of these factors is not quite clear; apparently, it may involve the strengthening of DC proteasome activity, induction of TAP protein transport to early endosomes, or enhancement of Ag transport from early endosomes to the cytosol [58].

In the normal state, cross-presentation in the absence of “danger signals” is an important mechanism for the induction and maintenance of tolerance to self Ags. For example, thymic CD8^+^ DCs have the ability of cross-presentation in normal conditions and participate in the destruction of developing autoreactive T cells [58].

**Presentation of lipid antigens by DCs with CD1 molecules**

Presentation of lipid Ags with CD1 molecules is a T cell stimulation pathway independent of MHC class I and class II molecules. CD1 proteins are structurally similar to MHC class I molecules, because they are heterodimers consisting of a CD1 heavy chain non-covalently bound to β2-microglobulin. Human DCs express five CD1 proteins: CD1a, CD1b, CD1c, CD1d, and CD1e, whereas mouse DCs express only one CD1d protein. The structure and function of these proteins have some differences, but their common main function is to present lipid antigens to T cells [59].

CD1a, CD1b, CD1c, and possibly CD1d are involved in the presentation of microbial lipid and lipopeptide antigens, such as mycolic acid, phosphatidylinositol mannoside, lipoarabinomannan, didehydroxy mycobactin, etc., to T cells. In addition, CD1d and, in some cases, CD1a, CD1b, and CD1c are able to present self lipid antigens. T cells recognize CD1-antigen complexes with T cell receptors (TCRs) that do not differ structurally from the TCRs interacting with MHC-antigen complexes. CD1-restricted TCRs that recognize foreign antigens are able to distinguish even small changes in the structure of a hydrophilic group of the lipid antigen [59].

The resulting T cells are involved in immune responses against bacterial (*Mycobacterium tuberculosis*,* Pseudomonas aeruginosa*, *Borrelia burgdorferi*, etc.), parasitic (*Leishmania major*, *Trypanosoma cruzi*,* Trypanosoma gondii*, etc.), viral (herpes simplex virus type 1 and 2, coxsackie virus b3, hepatitis B virus, etc.), and fungal (*Cryptococcus neoformans*) infections [59].

**Tumor escape from immune surveillance through the suppression of DC functions**

Many types of tumors are known to contain functionally abnormal DCs [60-62]. Furthermore, direct suppression of the proliferation and differentiation of T cells by a tumor or its environment or suppression of DC differentiation are considered to be an important mechanism of tumor escape from the immune system. In this part of the review, we describe the adverse effects of a tumor and its environment on the functional activity of DCs, leading to a suppression of the specific activation of effector CD4^+^ and CD8^+^ T cells.

**Tumor stroma**

An important component that ensures tumor resistance to the immune system is the tumor stroma. The stroma consists of fibroblasts, endothelial cells, and components of the extracellular matrix and inflammatory infiltrate. The latter is localized in the tumor stroma and consists of, in particular, myeloid-derived suppressor cells (MDSCs) and tumor-associated macrophages (TAMs) [63, 64]. Stromal cells produce a variety of factors, including cytokines, chemokines, growth factors, hormones, prostaglandins, lactic acid salts, and gangliosides, promoting the suppression of a DC-mediated response of effector CD4^+^ and CD8^+^ T cells and induction of Treg cells [65, 66]. In addition, direct chemical or enzymatic interactions between leukocytic products and clones of tumor-specific T cells have been reported, e.g. nitrotyrosination of T cell receptors and CD8 molecules, which led to the attenuation of antitumor T cell functions [67].

**Mechanisms of suppression of DC functions by tumor**

There are several mechanisms by which a tumor suppresses or even switches off DC functions. First, the tumor can prevent the penetration (infiltration) of DCs and pre-DCs into the tumor tissue. However, according to some reports, most tumors are infiltrated with even a higher number of DCs than normal tissues [68, 69]. This is due to the fact that tumor cells can produce chemokines, e.g. MIP-3α, that are “selectively chemotaxic” for immature DCs expressing the CCR6 receptor for MIP-3α [69].

Second, a tumor can suppress the maturation of infiltrating immature DCs, which may lead to the development of T cell tolerance. In fact, increased expression of co-stimulatory molecules by macrophages and DCs was found in the leukocytic infiltrate of certain tumors, but the ability of these DCs to present Ags was significantly reduced [61, 70].

Third, phagocytosis and processing of soluble tumor Ags in DCs can be suppressed or completely blocked. For example, a reduced efficiency of Ag uptake was observed in DCs derived from kidney cancer patients [68]. Inhibition of DC phagocytosis is often associated with secretion of the vascular endothelial growth factor (VEGF), which is one of the most important immunosuppressive cytokines produced by a tumor [71]. Several studies have demonstrated a relationship between an elevated VEGF level in the serum of cancer patients and the number and functionality of circulating DCs [72, 73]. Blockage of VEGF was found to increase Ag uptake and the migratory ability of tumor-specific DCs [71].

Fourth, DC migratory activity can be reduced, which is considered as another mechanism by which a tumor escapes the immune response [66]. Indeed, cytokines and growth factors such as IL-10, TGF-β, and VEGF [61] are overexpressed in tumor tissue just as the chemoattractant factors MIP-3α/CCL20 are [69]. On the other hand, tumor tissue contains factors such as gangliosides that inhibit DC migration. Both mechanisms can regulate the recruitment and migration of DCs into the tumor environment.

Fifth, the suppression of DC functions and tumor progression are affected by the inflammation that often accompanies malignancies [74]. Inflammatory mediators can be produced by both tumor cells and tumor stromal cells comprising various leukocyte subsets, in particular myeloid-derived suppressor cells [63] and tumor-associated macrophages [64]. Inflammatory mediators can cause leucopenia and affect angiogenesis and tumor cell survival, motility, and chemotaxis [75].

Overexpression of the STAT3 protein by tumor cells affects the expression of several immunosuppressive cytokines, including IL-10 and TGF-β, suppresses the Th1 response, reduces the expression of co-stimulatory molecules and MHC class II molecules, and activates TGF-β expression in DCs. Tumor progression also correlates with the accumulation of the immature DCs that induce Treg proliferation in tumor-infiltrated lymph nodes.

Sixth, the role of the tumor-secreted exosomes that mediate a variety of effects on immune competent cells, in particular on DCs, has been demonstrated [76, 77]. Exosomes of tumor cells are capable of suppressing the immune system through several mechanisms, including a reduction in the amount of DCs and suppression of their functions, attenuation of the proliferation and cytotoxicity of natural killer cells and T cells, and an increase in the amount of immunosuppressive cells (MDSC and Treg) [76, 77].

By affecting DCs, tumor exosomes facilitate an increase in STAT3 phosphorylation and IL-6 expression and, therefore, reduce both the activity and the number of DCs by inhibiting the differentiation of CD14^+^ monocytes into immature DCs. Furthermore, CD14^+^ cells in this case differentiate into HLA-DR^–/low^ cells that synthesize the TGF-β that inhibits T cell functions [76].

**The role of the tumor environment in the suppression of DC functions**

*Cytokines and growth factors associated with tumor progression. *The macrophage colony-stimulating factor (M-CSF) and IL-6 are important factors that are involved in the differentiation of monocytes [78, 79] and suppress the differentiation of DCs [80] by increasing the expression of M-CSF receptors in parallel with a reduction of the amount of GM-CSF α-receptors in pre- DCs. Similar phenomena are also characteristic of the IL-10 produced by tumor cells [81, 82]. *In vitro*, IL-10 inhibits the differentiation, maturation, and functional activity of DCs [83-85], switching the differentiation to mature macrophages [86].

Another growth factor secreted by various tumors under hypoxic conditions is VEGF. The VEGF level both in serum and in tumor tissue correlates with tumor progression [87, 88]. VEGF was shown to inhibit *in vitro *the development of DCs from CD34^+^ progenitors [89]. Furthermore, VEGF-exposed DCs reduced the production of IL-12, as well as the ability to stimulate allogeneic T cells [90]. VEGF inhibits the development of DCs, increasing the amount of immature myeloid cells [91].

*The effect of tumor-associated hypoxia on DC functions.* The tumor microenvironment is characterized by a low oxygen level (hypoxia) caused by reduced blood circulation in the tumor tissue [92]. Tumor hypoxia is associated with tumor progression, resistance to radioand chemotherapy [93], and macrophage phenotype changes [94, 95]. Under hypoxic conditions, DCs have a normal expression level of surface markers and cytokines, but the migration activity of DCs is inhibited [96, 97]. The physiological response to hypoxia is caused by the action of the hypoxia-induced factor (HIF) induced in the cell under hypoxic conditions [98, 99]. HIF targets include the genes encoding VEGF-A, glucose transporter 1 (Glut-1), and lactate dehydrogenase (LDH) [100]. A lactate dehydrogenase isoform, LDH-5, that transforms lactic acid into pyruvate at the lowest rate among enzymes of this type is not only overexpressed in various tumors, but is also associated with the aggressive phenotype of tumor cells [101]. A high expression of this isoenzyme leads to the accumulation of lactic acid in the tumor cells and microenvironment.

*The effect of an altered metabolism of tumor cells on DC functions. *The metabolism of tumor cells is well known to differ from that of normal cells. Tumor cells produce energy primarily through very active glycolysis, with subsequent formation of lactic acid, rather than through slow glycolysis and pyruvate oxidation in mitochondria using oxygen as in most normal cells. This phenomenon, called “aerobic glycolysis” or the “Warburg effect” (first described by Otto Warburg), leads to increased lactic acid production [102].

Tumors with a high level of lactic acid have an elevated lactate dehydrogenase level compared to that in normal tissue [103]; furthermore, the isoenzyme LDH-5 was detected in some tumors [101, 104]. A similar overexpression in non-small cell lung cancer or bowel adenocarcinoma is associated with an unfavorable prognosis [101, 104]. In 60–75% of colorectal cancer cases, high LDH-5 expression is strongly correlated with high expression of VEGF-R2 (KDR/Flk-1) [105]. Lactic acid is an important factor affecting DCs, which can facilitate tumor escape from the immune response.

Lactic acid has both negative and positive effects on the development of the T cell immune response [106, 107]. The sodium salt of lactic acid and glucose metabolites suppress the phenotypic and functional maturation of DCs, which correlates with the suppression of NF-κB activation [108]. Lactic acid induces changes in the expression of Ags in human monocyte-derived DCs and a decrease in the secretory capacity of DCs [109]. Lactic acid can also directly inhibit CD8^+^ T cells [110]. Extracellular acidosis leads to an accumulation of lactic acid in the tumor tissue. Several studies have described the adverse effects of acidic pHs on the functions of T cells and NK cells [111-113]. However, some researchers have noted an improved uptake of Ags by mouse DCs in acidosis and an increased efficiency of induction of specific CTLs [114].

Apart from lactic acid, other tumor cell metabolites can affect DC functions. The synthesis of arachidonic acid metabolites (prostanoids), including prostaglandin and thromboxane, is catalyzed by cyclooxygenases 1 and 2 (COX-1/2) [115]. The cyclooxygenase expression is altered in many tumors, e.g. colon, breast, lung, and ovarian cancers and melanoma [116-118]. COX-2 expression was found in tumor cells and tumor stroma cells [115]. In addition to a direct effect on tumor growth, apoptosis, cell-cell interactions, and angiogenesis, prostanoids suppress the antitumor immune response [118], in particular by inhibiting the differentiation and functions of DCs. For example, C. Sombroek *et al*. found an inhibitory effect of prostanoids and IL-6 on DC differentiation from CD34+ precursors and monocytes [119].

Gangliosides are tumor-cell-produced lipid derivatives that suppress the antitumor immune response [120-122] by inhibiting the differentiation of hematopoietic cells [120]. Some tumor types (neuroblastoma, retinoblastoma, melanoma, liver cancer, and colon cancer) and lymphomas are characterized by an anomalous ganglioside composition [123, 124], which may be associated with hypoxia [125]. Gangliosides impair the maturation and migration activity of Langerhans cells [126] and inhibit the differentiation, maturation, and functions of DCs [127].

## CONCLUSION

Knowledge about the origin and functions of dendritic cells, which has been accumulated over the past decade, has enabled the development of tumor immunology principles based on the involvement of body immune cells in fighting malignant diseases. However, many tumor types are associated with the suppression of the dendritic cells that are the most important immune system element that activates a specific antitumor response. This tumor escape of immune surveillance leads to a weakening of the components of both innate immunity (macrophages) and specific immunity (T cell elements). In this regard, it is evident that the development of DC-based antitumor vaccines should focus on the following issues: DC activation/maturation; the type of a tumor-specific antigen used to load dendritic cells; additional constructs encoding co-stimulatory molecules, to increase the efficiency of tumor antigen presentation; and methods of antigen delivery to dendritic cells which provide the highest level of processing and presentation of the antigen in complexes with MHC class I and class II molecules. Solving these problems will help develop protocols for the production of DC-based vaccines for an effective treatment of patients with various tumors.

## Abbreviations

Ag: antigen
APC: antigen-presenting cell
pDC: plasmacytoid dendritic cell
pre-DC: DC precursor
TCR: T cell receptor
Treg: regulatory T cell
CMP: common myeloid progenitor
HLA: human leukocyte antigen
MDP: macrophage-dendritic cell progenitor
MDSC: myeloid-derived suppressor cell;
NK: natural killer
TAM: tumor-associated macrophage
Th: T helper cell
TLR: toll-like receptor
low: low expression level
mid: middle expression level
high: high expression level
